# Inside-Out 3D Reversible Ion-Triggered Shape-Morphing Hydrogels

**DOI:** 10.34133/2019/6398296

**Published:** 2019-01-14

**Authors:** X. Du, H. Cui, Q. Zhao, J. Wang, H. Chen, Y. Wang

**Affiliations:** Institute of Biomedical & Health Engineering, Shenzhen Institutes of Advanced Technology (SIAT), Chinese Academy of Sciences (CAS), Shenzhen, 518035, China

## Abstract

Shape morphing is a critical aptitude for the survival of organisms and is determined by anisotropic tissue composition and directional orientation of micro- and nanostructures within cell walls, resulting in different swelling behaviors. Recent efforts have been dedicated to mimicking the behaviors that nature has perfected over billions of years. We present a robust strategy for preparing 3D periodically patterned single-component sodium alginate hydrogel sheets cross-linked with Ca^2+^ ions, which can reversibly deform and be retained into various desirable inside-out shapes as triggered by biocompatible ions (Na^+^/Ca^2+^). By changing the orientations of the patterned microchannels or triggering with Na^+^/Ca^2+^ ions, various 3D twisting, tubular, and plant-inspired architectures can be facilely programmed. Not only can the transformation recover their initial shapes reversibly, but also it can keep the designated shapes without continuous stimuli. These inside-out 3D reversible ion-triggered hydrogel transformations shall inspire more attractive applications in tissue engineering, biomedical devices, and soft robotics fields.

## 1. Introduction

Shape morphing has all-pervading presence in biological systems and is a critical aptitude for the survival of organisms. For example, in order to better adapt to the complex and ever-changing environment, many plants (e.g., Venus flytrap, mimosa, and pinecones) are able to change their shapes, of which the organs such as leaves, flowers, and tendrils respond to environmental stimuli (such as touch, light, or humidity) by varying internal turgor [1, 2]. It causes dynamic deformations due to the differences in local swelling behaviors, which are determined by anisotropic tissue composition and directional orientation of micro- and nanostructures within cell walls. Essentially, these deformations arise from the typical out-of-plane and in-plane gradient compositions or structures, or a combination of both, which amplify internal stresses under external stimuli [1, 3–5]. So far, scientists and engineers have been dedicated to the research of man-made, nonliving materials mimicking the behaviors that nature has perfected over billions of years.

Inspired by nature, efforts have been made on the exploration of various shape-morphing materials in the past decades, such as shape memory polymers, liquid crystal elastomers, and hydrogels [3, 6–12]. Among these, hydrogels represent one of the most promising candidates. Hydrogels are a class of three-dimensional (3D) networks formed by hydrophilic polymer chains embedded in a water-abundant environment, which can swell and shrink in response to certain stimuli, for example, light, pH, temperature, biochemical processes, and electric and magnetic fields. With the abilities to change their volumes sizably and reversibly, together with structures and functionalities comparable to biological systems, hydrogels favor competent applications in traumatic injuries, tissue engineering, and biosensors [13–17].

In recent years, various pathways for preparing shape-morphing hydrogels to mimic nature dynamic architectures have also been developed, which vastly broadens the application fields of hydrogels, including reconfigurable electronics, actuators, and soft robotics [18–22]. Their shape-morphing abilities are generally governed by nonuniform internal stresses led by uneven swelling/shrinking of different parts within a hydrogel sample. Therefore, a differentiated design at the molecular level (e.g., different swelling/shrinking properties design) and microscale (e.g., bilayer or patterned structure design) is necessary for designated deformations of the hydrogels. Conventionally, the shape-morphing hydrogels are prepared with different components across the hydrogel thickness (out-of-plane), for example, a bilayer design, which are prepared by combining two different layers with different swelling/shrinking rates or degrees [21, 23]. Recently, hydrogels with differential distributive components in-plane are also prepared via different approaches, for example, ionoprinting techniques, photopatterning techniques, and doped oriented fibers, which show in-plane differential responsiveness [3, 5, 10, 11, 19, 24–27]. However, none of these approaches employ a single and sustainable material which can transform controllably with the original 3D shape inside-out, nor do they obtain various reversible complex 3D shape-morphing hydrogels triggered by near-physiological stimuli.

Here, we demonstrate an intriguing strategy of combining the out-of-plane stress and in-plane stress for preparing 3D periodically patterned single-component polysaccharide (sodium alginate, SA) hydrogel sheets cross-linked with Ca^2+^ ions, which can precisely deform into designated shapes, as triggered by biocompatible ions (Na^+^/Ca^2+^). Due to being differentially cross-linked with Ca^2+^ ions, these hydrogels possess cross-linking density gradients both across the thickness (out-of-plane) and at the bottom surface (in-plane) via heterogeneous design by replicating microchannels at the bottom surfaces of the hydrogel sheets. Upon swelling or shrinking, the differential volume and out-of-plane stresses across the thickness cause bending deformations, while in-plane heterogeneity also leads to modulated internal stresses, resulting in controllable 3D deformations in water or calcium chloride (CaCl_2_) or sodium chloride (NaCl) solutions. Due to the cooperative effects, the deformation can be facilely programmed by changing the microchannel orientations. Therefore, tube-curling, twisting, and rolling deformations can easily be customized. By releasing part of the cross-linker Ca^2+^ ions with Na^+^ ions, the 3D hydrogel sheets can alter their shapes from 3D to 2D, even to complete inside-out 3D structures. Furthermore, various cooperative deformations and complex configurations can be obtained by combining differently oriented microchannels into one side of the hydrogel sheet, for example, “T”- or “H”-shape tubular structures, double helix and torsional helix structures, and various plant-inspired architectures. With an understanding on the basic design rules and robust strategies for cooperative deformations, a broad range of responsive materials can be employed and miniaturized to micrometer scales for more advanced and potential applications in tissue engineering, biomedical devices, and soft robotics fields.

## 2. Results

### 2.1. Fabrication of Patterned Hydrogels and Their Deformations

**Figure 1:**
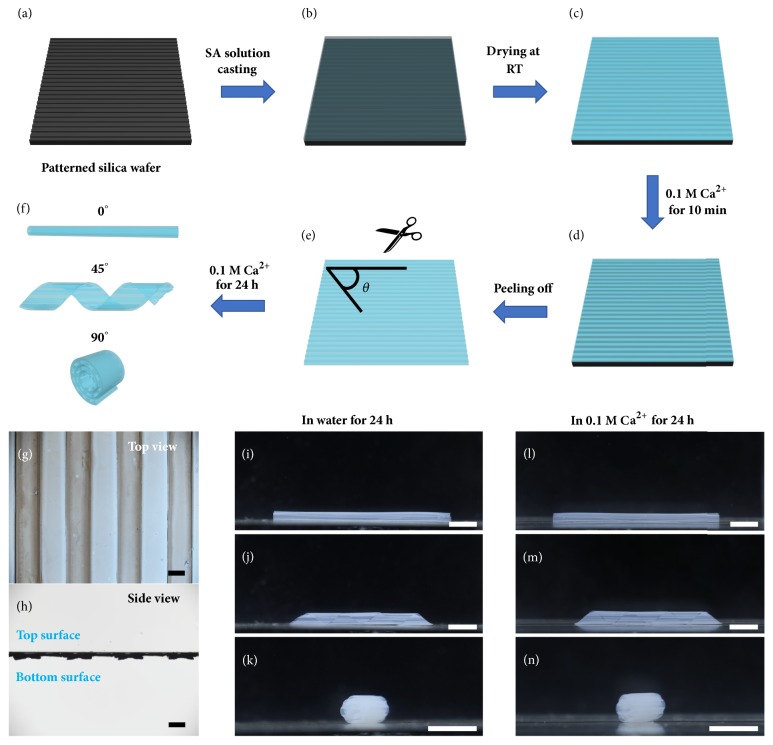
*Fabrication and shape deformation of patterned hydrogels.* (a) A patterned silica wafer with microchannel structures (width: 800 *μ*m; spacing: 800 *μ*m; height: 100 *μ*m) was fabricated by photolithography. (b) A SA pregel solution was casted onto the silica wafer and dried at room temperature (c). (d) The patterned hydrogel on the silica wafer was first immersed into a 0.1 M CaCl_2_ solution for 10 min, then cut with microchannels in different orientations after peeling off from the patterned silica wafer (e), and finally immersed into a 0.1 M CaCl_2_ solution for another 24 h (f). (g) and (h) show the top view and side view of the patterned hydrogel sheet. (i)-(n) show the 3D deformation of the resulting hydrogel sheets with tube-curling structures, helical structures, and rolling structures in the 0.1 M CaCl_2_ solution and water for 24 h, respectively, with alignment of microchannels at angles *θ* = 0° (i, l), 45° (j, m), and 90° (k, n) correspondingly. The scale bars are 0.5 cm.

In a typical experiment, the periodical patterned hydrogel films were fabricated by introducing well-aligned microchannels on one side of the films, as shown in Figures 1(a)–1(f). Firstly, these microchannel structures were fabricated by photolithography on the surface of silica wafers (fig. [Supplementary-material supplementary-material-1], [Supplementary-material supplementary-material-1]). These periodical patterns endowed the whole surface with well-aligned microchannels (width: 800 *μ*m; spacing: 800 *μ*m; height: 100 *μ*m), which facilitated SA (an anionic polysaccharide) pregel solution infiltration. After the pregel solution had filled all the channels by capillary force, it was cured to form patterned hydrogel films by water evaporation (fig. [Supplementary-material supplementary-material-1]). Then, the patterned hydrogels on the templates were first immersed into a 0.1 M CaCl_2_ solution for 10 min, where the SA was pre-cross-linked to form ionic cross-links with Ca^2+^ ions (fig. [Supplementary-material supplementary-material-1], [Supplementary-material supplementary-material-1]). Afterwards, microchannel-structured hydrogel films were obtained by peeling off from the silica templates (Figures 1(g)-1(h)). Finally, the hydrogel sheets were cut with microchannels in different orientations and further immersed into the 0.1 M CaCl_2_ solution for another 24 h to cross-link thoroughly, enabling controllable 3D transformation of hydrogel structures with microchannels facing inward (fig. [Supplementary-material supplementary-material-1], [Supplementary-material supplementary-material-1]). This shape transformation is caused by the large shrinking of the hydrogel at both the top surface and bottom surface in the previous cross-linking process.

The 3D shape-morphing degree of hydrogels depends on the pattern width (width: spacing = 1:1), pre-cross-linking time, and hydrogel thickness. Specifically, increasing the pattern width from 200 *μ*m to 3200 *μ*m decreases the bending degree of the resulting rolling structure from 2430°±28.6° to 1080°±27.5° correspondingly. The spacing in between the microchannels has an effect on the elastic tensors and, thus, on the deformation [28]. It clarifies that increasing the width of the microchannels decreases the tightness and smoothness of the rolling structures (fig. [Supplementary-material supplementary-material-1]-[Supplementary-material supplementary-material-1], [Supplementary-material supplementary-material-1]). In addition, the rolling tightness can also be tuned by extending the pre-cross-linking time from 10 min to 1440 min, leading to a decrease of the bending degree of the resulting rolling structures from 1980°±19.8° to 1080°±20.5° owing to the variation of mechanical properties (fig. [Supplementary-material supplementary-material-1]-[Supplementary-material supplementary-material-1], [Supplementary-material supplementary-material-1]). Moreover, by changing the thickness of the hydrogel sheets, the rolling degree can also be tuned due to the alteration of bending resistance, thus varying the rolling structures (fig. [Supplementary-material supplementary-material-1]-[Supplementary-material supplementary-material-1], [Supplementary-material supplementary-material-1]) [19].

The direction of the hydrogel deformation can be programmed by cutting the alignment of microchannels at a specific angle *θ* (Figures 1(i)–1(n)). With microchannels aligned at *θ* = 0°, 45°, or 90°, the resultant SA hydrogel sheets were imparted with a tube-curling structure, a helical structure, or a rolling structure correspondingly, and the deformed structures show no significant differences in water (Figures 1(i)–1(k)) and in the 0.1 M CaCl_2_ solution (Figures 1(l)–1(n)) due to no differences in osmotic pressure. Figures 1(i)–1(n) show that all these bending directions are parallel to the microchannel orientations. The design of microchannel structures enables the programmed deformations and yet reduces the mechanical properties of the hydrogels accordingly. fig. [Supplementary-material supplementary-material-1] illustrates engineering force-strain curves of the hydrogels. The results indicate that the well-designed microchannel structures and larger cutting angle lead to decreases in the mechanical properties.

### 2.2. Mechanism of Programmed 3D Deformations

To understand the mechanism of the programmed 3D deformations of the hydrogels, hydrogel sheets solely with a cross-linking density gradient or well-aligned microchannels were fabricated, respectively (fig. [Supplementary-material supplementary-material-1]). It is notable that the hydrogel deforms randomly when there is only cross-linking density gradient across the thickness (fig. [Supplementary-material supplementary-material-1]). Similarly, the hydrogel sheet with microchannel structures but no cross-linking density gradient at the bottom surface also randomly deforms (fig. [Supplementary-material supplementary-material-1]), resulting from the lower cross-linking density in the middle layer of the hydrogel (fig. [Supplementary-material supplementary-material-1]). This implies that a designated 3D deformation cannot be obtained for SA hydrogels solely with the cross-linking density gradient or well-aligned microchannel structures. Based on this evidence, it can be concluded that only a combination of both a cross-linking density gradient across the thickness and well-aligned microchannels could demonstrate controllable and precise deformation in a desirable manner (Figures 1(i)–1(n)) due to the cooperative out-of-plane and in-plane stress.

**Figure 2:**
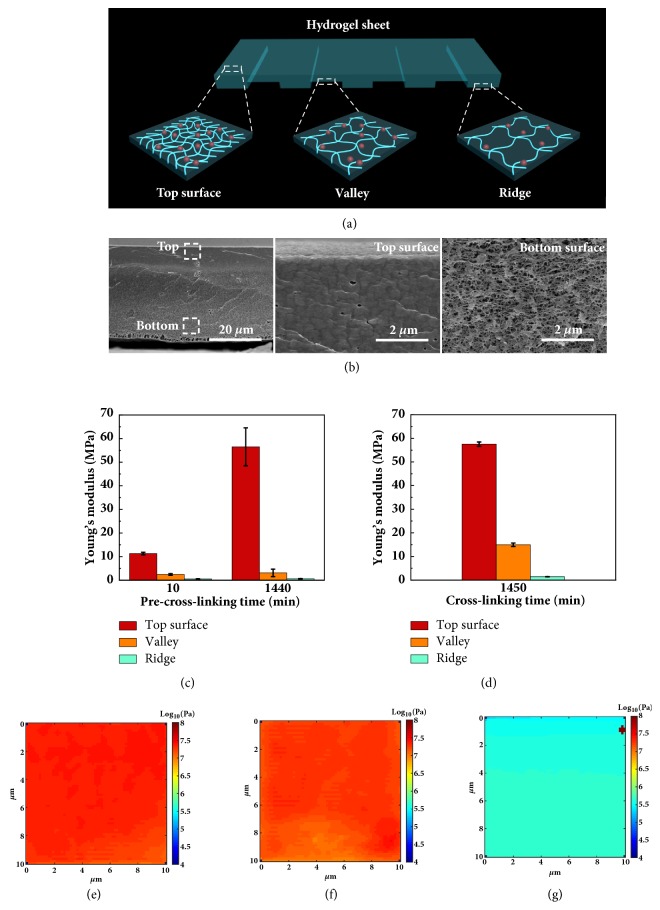
*Schematic illustrations and mechanical analyses of the programmed 3D deformations.* (a) The scheme shows the differences of the Ca^2+^ ions diffusion and cross-linking density at different areas of the hydrogel sheet. (b) The cross-sectional field-emission-scanning electron microscopy (FE-SEM) images of the frozen dried hydrogel and the magnified images of the top surface and the bottom surface. (c) Increasing the pre-cross-linking time increases Young's moduli at the three representative areas of the hydrogel sheet. (d) Young's modulus decreases across the thickness from the top surface of the hydrogel sheet, which is 57.55±0.91 MPa for the top surface, 14.94±0.73 MPa for the valley, and 1.42±0.038 MPa for the ridge, respectively. (e) Young's modulus mechanical maps for the top surface, valley (f), and ridge (g), respectively.

To further investigate the mechanism of the 3D deformations, the mechanical properties at different parts of hydrogel sheets with both the cross-linking density gradient and microchannel structure design were analyzed.  Figure 2(a) illustrates that the amount of Ca^2+^ ions diffused into the SA hydrogel network determines the network density in the pre-cross-linking process, in which the SA films are still attached on the patterned silica wafer [29, 30]. Thus, the SA chains form the tightest networks at the top surface due to their complete exposure to the Ca^2+^ reservoir, resulting in the highest cross-linking density. In comparison, the SA chains form looser networks at the ridges and valleys due to decreasing Ca^2+^ concentration diffusible into the deeper parts of the hydrogel sheet, thus causing lower cross-linking density. After the pre-cross-linking process, the SA films were peeled off from the patterned silica wafer and immersed into the 0.1 M CaCl_2_ solution for further cross-linking. However, a further cross-linking time of 24 h only increases the overall Young's moduli of the hydrogel, and the differences of cross-linking density across the thickness of the hydrogel sheet still exist due to their variation of networks in the pre-cross-linking process (Figures 2(b)–2(d), fig. [Supplementary-material supplementary-material-1]).  Figure 2(b) demonstrates that the hydrogel networks become looser from the top surface to the bottom surface, strongly indicating the cross-linking density gradient across the thickness even after a further cross-linking time of 24 h. Figures 2(e)–2(g) further experimentally verify that Young's modulus decreases across the thickness of the hydrogel sheet, which is 57.55±0.91 MPa for the top surface (Figure 2(e)), 14.94±0.73 MPa for the valleys (Figure 2(f)), and 1.42±0.038 MPa for the ridges (Figure 2(g)), respectively. The gradual decrease in Young's modulus across the thickness of the hydrogel sheet brings about an out-of-plane stress in the swelling or shrinking process. Meanwhile, Young's moduli are also different at the bottom surface between the ridges and the valleys, which results in an in-plane stress during the swelling or shrinking process. Therefore, the combination of dual cross-linking density gradients both across the thickness and at the bottom induces a cooperative effect between out-of-plane and in-plane stresses during the swelling or shrinking process, leading to designated shape transformations of the hydrogel sheets (fig. [Supplementary-material supplementary-material-1]). The bending energy is from the out-of-plane stress, while the bending direction is determined by the in-plane stress (the orientations of the microchannels).

### 2.3. Tunable Actuation of Programmed Hydrogels

**Figure 3:**
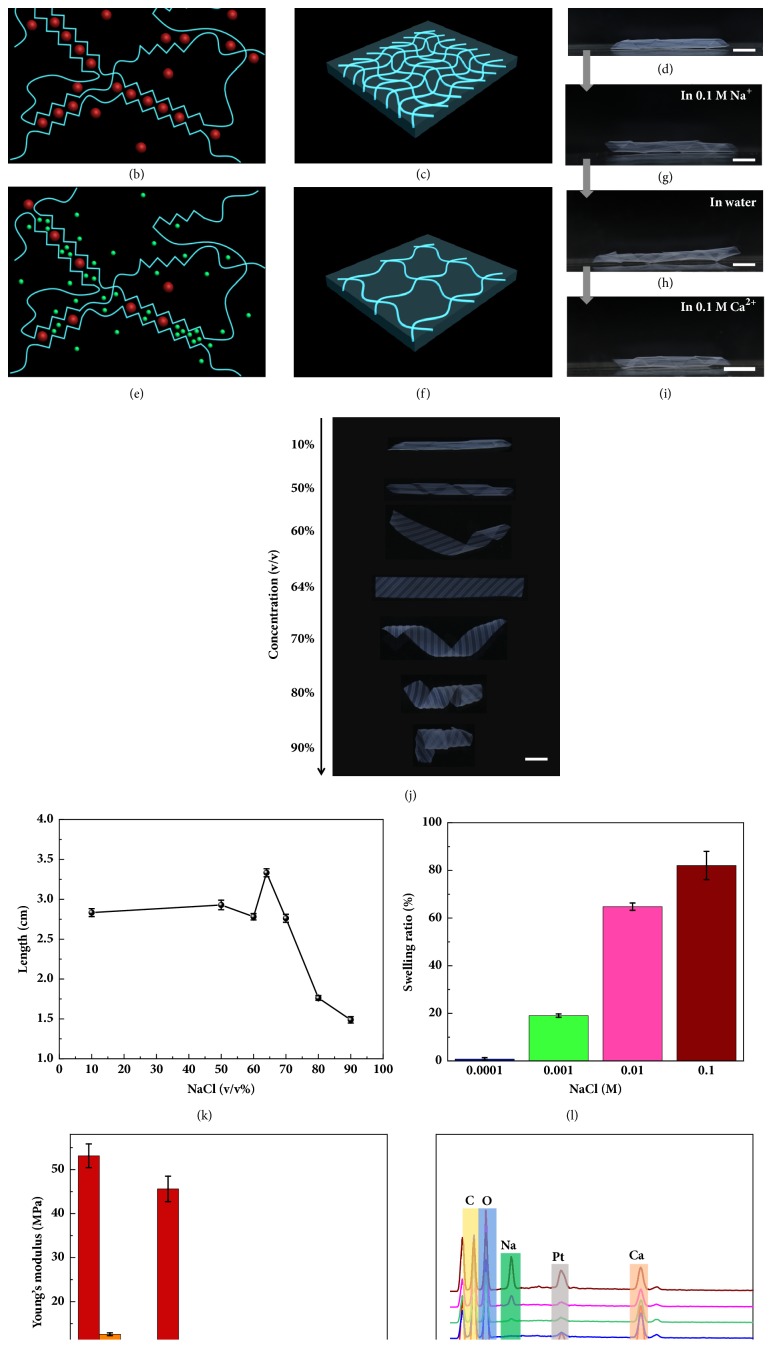
*Tunable actuation of programmed hydrogels.* (a) In the SA hydrogel, the G-blocks and MG-blocks on polymer chains form ionic cross-links through Ca^2+^ (b), resulting in tight polymer chains in the CaCl_2_ solution (c). (e) Partial ionic cross-links are unlocked as certain amounts of Ca^2+^ ions are replaced by a large amount of Na^+^ ions, resulting in looser polymer chains in the NaCl solution (f). (d) shows the helical structure of the hydrogel sheet with microchannels facing inward in the 0.1 M CaCl_2_ solution, which changes its helical rotation oppositely with microchannels facing outward in the 0.1 M NaCl solution (g). (h) The secondly deformed helix can retain its shape in water or recovers to its primary helix with microchannels facing inward again after being immersed in the 0.1 M CaCl_2_ solution (i). (j) The images of dynamic deformation of hydrogel sheets in a mixed solution of 0.001 M NaCl and 0.001 M CaCl_2_ for 24 h and the corresponding curve (k). (l) Increasing the concentration of NaCl changes the length of the hydrogel sheet due to the swelling ratio changes in various NaCl solutions (0.0001 M, 0.001 M, 0.01 M, and 0.1 M). (m, n) The EDS demonstrates the change of Na^+^ and Ca^2+^ ions in various conditions. The scale bars are 0.5 cm.

To achieve effective reversibility and for a more sophisticated manipulation of the cooperative deformations, a Na^+^/Ca^2+^ cations system was employed (Figure 3). In water or the CaCl_2_ solution, the cross-linked hydrogels appeared as a helical structure with the bottom surface of ridges and valleys facing inward (Figures 1(j), 1(m), and 3(d), fig. [Supplementary-material supplementary-material-1], [Supplementary-material supplementary-material-1]). After immersing the hydrogel sheet into the 0.1 M NaCl solution, interestingly, a secondary deformation occurred in which the helix turned its helical rotation oppositely with the ridges and valleys facing outward (Figure 3(g), fig. [Supplementary-material supplementary-material-1], [Supplementary-material supplementary-material-1]). It takes only 30 min to complete the actuation process, and the secondary deformed helical structure can retain its shape in both the 0.1 M NaCl solution and water (Figures 3(g) and 3(h), [Supplementary-material supplementary-material-1]). After immersing back into the 0.1 M CaCl_2_ solution, the oppositely rotated helical structure can recover its primary deformed shape with the ridges and valleys facing inward again in less than 1 min (Figure 3(i), [Supplementary-material supplementary-material-1]). The hydrogels preserve excellent performance even after more than 10 cycles of the reversible shape transformations via immersing into the 0.1 M CaCl_2_ and 0.1 M NaCl solution repeatedly back and forth (fig. [Supplementary-material supplementary-material-1]). This tunable actuation implies that the presence of Na^+^ ions will influence the ionic cross-linkages between COO^−^ and Ca^2+^, thus changing the swelling or shrinking properties and causing an inside-out transformation of the programmed hydrogels.

As previously reported, alginates are linear polysaccharides consisting of 1→4 linked *β*-D-mannuronic acid (M) and *α*-L-guluronic acid (G) which are comprised of sequences of M (M-blocks) and G (G-blocks) residues interspersed with MG sequences (MG-blocks). Gelation is driven by the interactions between G-blocks which associate to form tightly held junctions in the presence of Ca^2+^ ions. In addition to G-blocks, MG-blocks also participate, forming weak junctions (Figures 3(a)–3(c)) [29, 30]. Based on this, our hypothesis is that these junctions between COO^−^ and Ca^2+^ can be broken by Na^+^ ions, where Na^+^ ions diffuse into the hydrogel and compete with Ca^2+^ ions to weaken the ionic cross-links, thus releasing the Ca^2+^ ions from the hydrogel (Figures 3(e) and 3(f)) [31]. To further verify our hypothesis, the deformed hydrogel sheet with helical structure was immersed into a mixed solution of 0.001 M NaCl and 0.001 M CaCl_2_. Figures 3(j) and 3(k) show that when the concentration of NaCl increases from 10 v/v% to 60 v/v%, the helix sheet becomes looser due to the replacement of Ca^2+^ ions by the competing Na^+^ ions which causes a decrease in cross-linking density and an increase in the swelling ratio of the hydrogel. The helical sheet becomes flattened when the concentration of NaCl is 64 v/v%. For a further increase in the concentration of NaCl to 90 v/v%, when more Ca^2+^ ions are replaced by Na^+^ ions and the cross-linking density is lowered, the secondary deformation occurs in which the ridges and valleys turn to be facing outward. Additionally, the bending directions of the helical sheet also change from parallel to perpendicular to the microchannel orientations. With an increase in the initial concentration of the mixed solution for comparison (0.1 M NaCl and 0.1 M CaCl_2_), the helical sheet turns to be flat when the concentration of NaCl is as high as 98 v/v% because of a sharp increase in the Ca^2+^ ions concentration of the mixed solution (fig. [Supplementary-material supplementary-material-1]). Figure 3(l) further confirms that the swelling ratio increases with the NaCl concentration from 0.0001 M to 0.1 M. Accordingly, Young's moduli of the top surfaces decrease sharply from 53.15 MPa to 1.31 MPa with an increase of Na^+^ concentration from 0.0001 M to 0.1 M, indicating that more Ca^2+^ ions are released from the hydrogel (Figure 3(m)). In addition, we further verify our hypothesis with an energy dispersive spectrometer (EDS) to demonstrate the change of Na^+^ and Ca^2+^ ions in various conditions. Figure 3(n) shows that there is a Na peak in the SA without any treatment but no Ca peak. After the hydrogel cross-linked with 0.1 M CaCl_2_ for 24 h, the Na peak disappears while the Ca peak arises, showing that the free Na^+^ ions are replaced by Ca^2+^ ions. When the cross-linked hydrogel is further immersed in various NaCl solutions (0.0001 M, 0.001 M, 0.01 M, and 0.1 M) for 24 h, respectively, the Na peak increases again while the Ca peak decreases, indicating that more Ca^2+^ ions are released from the hydrogel. Thus, the hydrogel sheets change their helical rotation oppositely due to a larger swelling ratio at the bottom surface compared to the top surface, where the ionic cross-links decrease with the increasing concentration of Na^+^ ions.

### 2.4. Multiple Cooperative Shape Deformations

**Figure 4:**
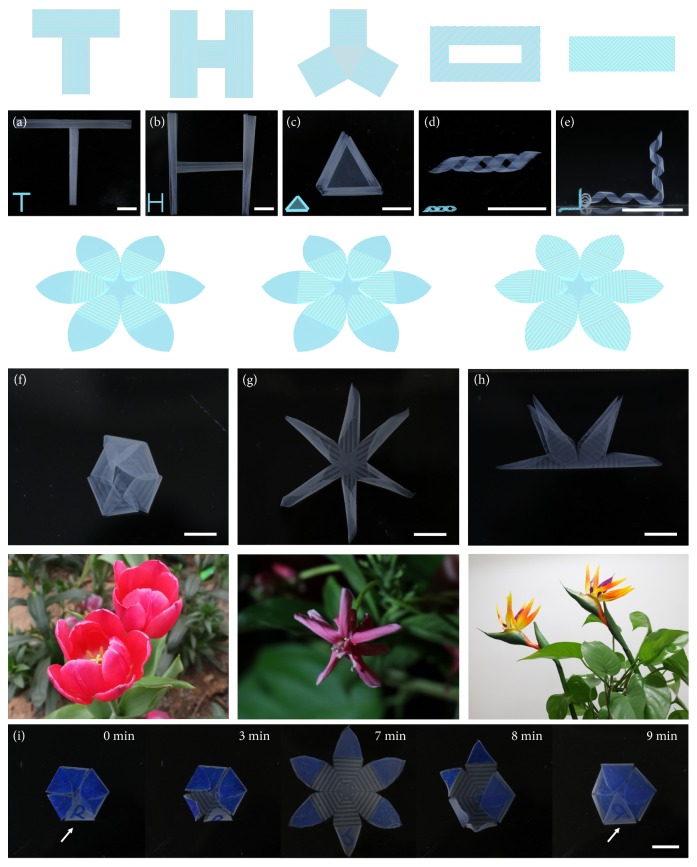
*Cooperative shape deformations.* (a) Connected “T” tube in the 0.1 M CaCl_2_ solution with combined alignment of microchannels at angles* θ *= 0° and* θ *= 90°. (b) Connected “H” tube in the 0.1 M CaCl_2_ solution with a combined alignment of microchannels at angles* θ *= 0° and* θ *= 90°. (c) Connected triangular tube in the 0.1 M CaCl_2_ solution with combined alignment of microchannels at angles* θ *= 0°,* θ *= 60°, and* θ *= 120°. (d) A double helix in the 0.1 M CaCl_2_ solution with alignment of microchannels at angle* θ *= 45°. (e) Connected torsional helix structure in the 0.1 M CaCl_2_ solution with a combined alignment of microchannels at angles* θ *= 45° and* θ *= 135°. (f) Various artificial six-petal flower structures in the 0.1 M CaCl_2_ solution comprised of microchannels aligned at 0°, 90° (g) and 0°/90° (h). (i) shows the dynamic opening and closing processes of an artificial flower in the 0.1 M NaCl solution, where the petals were partially coated with a blue dye to demonstrate the dynamic inside-out shape transformations of the hydrogels. The scale bars are 1 cm.

By the combination of differently aligned microchannels that generate simple deformations, we design and create a series of complex 3D architectures based on the art of “kirigami.” Starting from the simplest structures, a combination of microchannels aligned at 0°, 90°, and 0°/60°/120° configurations in one hydrogel sheet gives rise to T-shaped, H-shaped, and triangular hydrogel tubes in good shapes (Figures 4(a)–4(c)). For a more advanced deformation, a perfect double helix, which is comparable to the typical DNA molecular configurations, can be generated by a hollow rectangular hydrogel sheet with microchannels aligned at 45°. In addition, oriented microchannels with 45°/135° configurations in a hydrogel sheet result in a torsional helical structure (Figures 4(d) and 4(e)). All the deformations are parallel to the orientation of microchannels (fig. [Supplementary-material supplementary-material-1]), which show good predictability and stability to be applied in 3D scaffolds for tissue engineering, 3D microfluidics, or microswimmers.

Inspired by various flowers, we further designed artificial six-petal flower structures comprised of oriented microchannels with 0°, 90°, and 0°/90° configurations.  Figure 4(f) shows that a bud structure can be fabricated by aligning microchannels at 0° at the lower half of each petal. Thus, each petal can “close” parallelly to the orientation of microchannels, which gives the shape of a bud.  Figure 4(g) illustrates an artificial blooming flower, in which the microchannels aligned at 90° at the lower half of each petal. With a combination of these two designs, an interesting structure comparable to a bird-of-paradise flower is vividly demonstrated, in which the microchannels are aligned at 0° at the lower half of each petal and 90° at the upper half of each petal (Figure 4(h)). Furthermore, we also mimicked the flower opening and closing processes by treating the previous six-petal flower structure with 0.1 M Na^+^ ions, where five petals were partially coated with blue dye and one petal was written with the letter “R” on the nonmicrochannel surface. The closed petals with the letter “R” outside, in which the microchannels aligned at 0°, opened slowly after immersing in the 0.1 M Na^+^ solution for 3 min. After 7 min, the flower opened completely into a flower. Further increasing the immersion time to 9 min, the flower closed oppositely into a complete bud with the letter “R” inside (Figure 4(i)). By immersing the six-petal flower in the 0.1 M NaCl solution, the different stages of an opening flower can be vividly demonstrated, and the letter “R” on the petal indicates the inside-out shape transformations of the hydrogels.

## 3. Discussion

Based on the hydrogel shape-morphing behaviors with different microchannel alignments demonstrated in the previous examples, our model provides a simple strategy for the design of predictable, controllable, and reversible 3D transformation of 2D materials. More importantly, the results arouse inspirations for the design and application of shape-morphing materials to meet specific targets and standards. As an unprecedented attempt, we demonstrate the possibility of enabling controllable and precise deformation in a desirable manner using a single component of a natural polysaccharide with convenient and nontoxic ions stimuli (Na^+^/Ca^2+^). Simply by varying the concentration of Na^+^ or Ca^2+^ ions, the fabricated hydrogels are able to transform spontaneously between the shapes of a complex 3D structure and 2D planar architecture. The good agreement among the dynamic 3D shape morphing, morphological gradient, Young's modulus variation, and change of Na^+^/Ca^2+^ in hydrogels verifies the hypothesis that ionic cross-links with Ca^2+^ can be released by Na^+^ ions of certain concentration and thus cause secondary transformation with good reversibility.

Our ion-triggered shape-morphing strategy relies on a combination of out-of-plane and in-plane stresses resulting from the cross-linking density gradients across the thickness and at the bottom surface of the hydrogel during the swelling or shrinking process. This strategy could be a platform technology which is applicable to not only the demonstrated hydrogels but also a broad range of polymers (for example, UV-polymerized materials, other ionic-cross-linked hydrogels). Through the control of deformation parameters, which are as simple as pattern width, pre-cross-linking time, hydrogel sheet thickness, microchannel orientation, ionic concentration, and ion-triggering time, we can create various architectures and demonstrate programmable deformations comparable to the flowering process by immersion in certain ionic solutions. Due to the employed single-component material, our system can be extended to a multicomposite or multifunction system for fabricating various architectures with specific properties or functions via introducing other components or sensing functionalities (for example, temperature sensing, photo sensing, and magnetic sensing) into this system [9, 19, 32]. Owing to our biocompatible and sustainable hydrogels together with the triggering method of near-physiological stimuli, our study opens new avenues for creating desirable shape-morphing architectures for tissue engineering, biomedical devices, soft robotics, and beyond.

## 4. Materials and Methods

### 4.1. Materials

Sodium alginate (SA) was purchased from Sigma Aldrich. Sodium chloride and calcium chloride were obtained from Sinopharm Chemical Reagent Co., Ltd. SU-8 3050 and SU-8 developer were purchased from MicroChem Corporation. All reagents were used as received. Ultra-pure water was used in all the experiments.

### 4.2. Fabrication of Microchannel-Patterned Silica Wafers

Various microchannels with 100 *μ*m height, different spacings (spacing of 200 *μ*m, 400 *μ*m, 800 *μ*m, 1600 *μ*m, and 3200 *μ*m, width: spacing = 1:1), and combined orientations (0°, 45°, 90°, 0°/90°, 45°/135°, and 0°/60°/120°) on the silica wafers were prepared, respectively, by photolithography (EVG 610, Austria) according to our previous work [33]. Before use, all the patterned silica wafers were treated with oxygen plasma (Weike PDC-M, China) [34].

### 4.3. Fabrication of SA Hydrogels with Various Programmed Deformations

A 1.0 mL SA pregel solution (5.0 wt %) was poured into a cell composed of a microchannel-patterned silica wafer and a 1 mm thick silicone rubber spacer and then evaporated at room temperature for 5 h. Afterwards, the SA-casted silica wafer was immersed into the 0.1 M calcium chloride solution for 10 min, forming a SA hydrogel sheet (4.0 cm × 4.0 cm, 200.0 *μ*m in thickness) with well-aligned microchannels (800 *μ*m in width, 800 *μ*m in spacing, and 100 *μ*m in height). Then, the obtained SA hydrogel sheet was first cut into various stripes (3.5 cm × 0.5 cm) with cutting angles of 0°, 45°, and 90°, respectively, along the axis of patterns and then completely cross-linked in the 0.1 M CaCl_2_ solution for another 24 h. The SA hydrogel was washed with extensive ultra-pure water to remove the residual Ca^2+^ ions for further use. Unless otherwise specified, the hydrogel sheets with different cutting angles or complex configurations were prepared in the same manner as mentioned above.

### 4.4. Fabrication of SA Hydrogels with Various Widths of Microchannels

A 1.0 mL SA pregel solution (5.0 wt %) was poured into a cell composed of a patterned silica wafer and a 1 mm thick silicone rubber spacer and then evaporated at room temperature for 5 h. Afterwards, the SA-casted silica wafers were immersed into the 0.1 M calcium chloride solution for 10 min, forming a SA hydrogel sheet (4.0 cm × 4.0 cm, 200.0 *μ*m in thickness) with various widths of well-aligned microchannels (200 *μ*m, 400 *μ*m, 800 *μ*m, 1600 *μ*m, and 3200 *μ*m in width; 100 *μ*m in height; width: spacing = 1:1), respectively. Then, the obtained SA hydrogel sheets were first cut into stripes (3.5 cm × 0.5 cm) with a cutting angle of 90°, respectively, along the axis of patterns and then completely cross-linked in the 0.1 M CaCl_2_ solution for another 24 h.

### 4.5. Fabrication of SA Hydrogels with Different Cross-linking Times

A 1.0 mL SA pregel solution (5.0 wt %) was poured into a cell composed of a microchannel-patterned silica wafer and a 1 mm thick silicone rubber spacer, respectively, and then evaporated at room temperature for 5 h. Afterwards, the SA-casted silica wafer was immersed into the 0.1 M calcium chloride solution for various pre-cross-linking times of 10 min, 2 h, 4 h, 8 h, 16 h, and 24 h, respectively, forming a SA hydrogel sheet (4.0 cm × 4.0 cm, 200.0 *μ*m in thickness) with well-aligned microchannels (800 *μ*m in width, 800 *μ*m in spacing, and 100 *μ*m in height). Then, the obtained hydrogel sheet was first cut into a stripe (3.5 cm × 0.5 cm) with a cutting angle of 90° along the axis of patterns and then completely cross-linked in the 0.1 M CaCl_2_ solution for another 24 h.

### 4.6. Fabrication of SA Hydrogels with Various Thicknesses

A SA pregel solution (5.0 wt %) with a certain volume (0.5 mL, 1.0 mL, 1.5 mL, 2.5 mL, and 2.0 mL) was poured into a cell composed of a microchannel-patterned silica wafer and a 1 mm thick silicone rubber spacer, respectively, and then evaporated at room temperature for 5 h. Afterwards, the hydrogel sheet (4.0 cm × 4.0 cm, 142 *μ*m, 200 *μ*m, 257 *μ*m, 300 *μ*m, and 343 *μ*m in thickness) was peeled off from the silica wafer. Then, the obtained hydrogel sheet was first cut into a stripe (3.5 cm × 0.5 cm) with a cutting angle of 90° along the axis of patterns and then completely cross-linked in the 0.1 M CaCl_2_ solution for 24 h.

### 4.7. Fabrication of SA Hydrogels with Solely Cross-linking Density Gradient

A 1.0 mL SA pregel solution (5.0 wt %) was poured into a cell composed of a smooth silica wafer and a 1 mm thick silicone rubber spacer and then evaporated at room temperature for 5 h. Afterwards, the SA-casted silica wafer was immersed into the 0.1 M calcium chloride solution for 10 min, forming a SA hydrogel sheet (4.0 cm × 4.0 cm, 200.0 *μ*m in thickness). Then, the obtained SA hydrogel sheet was first cut into a stripe (3.5 cm × 0.5 cm) with a cutting angle of 90° along the axis of the patterns and then completely cross-linked in the 0.1 M CaCl_2_ solution for another 24 h.

### 4.8. Fabrication of SA Hydrogels with Only Well-Aligned Microchannels

A 1.0 mL SA pregel solution (5.0 wt %) was poured into a cell composed of a microchannel-patterned silica wafer and a 1 mm thick silicone rubber spacer and then evaporated at room temperature for 5 h. Afterwards, the hydrogel sheet (4.0 cm × 4.0 cm, 200.0 *μ*m in thickness) was peeled off from the silica wafer. Then, the obtained hydrogel sheet was first cut into a stripe (3.5 cm × 0.5 cm) with a cutting angle of 90° along the axis of patterns and then completely cross-linked in the 0.1 M CaCl_2_ solution for 24 h.

### 4.9. Tunable Actuation of Programmed Hydrogels

To study the shape-morphing differences of the hydrogel sheets with oriented microchannels at 45° (3.5 cm × 0.5 cm, 200.0 *μ*m in thickness), the hydrogels were immersed into various mixed solutions (0.001 M NaCl with 10 v/v%, 20 v/v%, 30 v/v%, 40 v/v%, 50 v/v%, 60 v/v%, 64 v/v%, 70 v/v%, 80 v/v%, and 90 v/v% by volume in 0.001 CaCl_2_ and 0.1 M NaCl with 10 v/v%, 20 v/v%, 30 v/v%, 40 v/v%, 50 v/v%, 60 v/v%, 70 v/v%, 80 v/v%, 90 v/v%, and 98 v/v% by volume in 0.1 M CaCl_2_) for 24 h. To study the actuation of the hydrogel sheets with oriented microchannels at 45°, the cross-linked hydrogels were immersed into the 0.1 M NaCl aqueous solution for 30 min and then transferred into the 0.1 M CaCl_2_ solution for 1 min. The reversibility performance was evaluated by repeating the above steps 10 times. Before the switch of immersing solutions, all the former SA hydrogels were washed with extensive ultra-pure water to remove the residual Ca^2+^ or Na^+^ ions for the following experiments. The hydrogel swelling ratio based on mass was calculated by dividing the gel mass after swelling by the initial gel mass.

### 4.10. Characterization of SA Hydrogels

The morphologies and sizes of the silica wafer and SA hydrogels were determined by an optical microscope (Nikon Ni-U, Japan) and a field-emission-scanning electron microscopy (FE-SEM, ZEISS SIGMA 300). The mechanical properties of the hydrogels were determined on a testing machine (Instron, Legend 2344). All the shape-morphing behaviors of the hydrogels were recorded by a digital camera (Canon, 7D Mark II). The element compositions of SA hydrogels were detected by an energy dispersive spectrometer analyzer (EDS, X-MaxN). The local elasticities of the SA hydrogel samples were probed with a commercial atomic force microscope (AFM, Dimension Icon, Bruker) in force volume (FV) mechanical imaging mode according to our previous work [19]. An average elasticity was obtained via measuring five different areas of the SA hydrogel samples.

## Data Availability

All data needed to evaluate the conclusions in the paper are present in the paper and/or Supplementary Materials. Additional data related to this paper may be requested from the authors.

## Supplementary Materials

Supplementary Materials fig. S1: images of patterned silica wafer and sodium alginate hydrogel. fig. S2: the influence of Ca^2+^ ions concentration on the gelation process. fig. S3: investigation of the parameters involved in programmed deformations. fig. S4: mechanical properties of hydrogels. fig. S5: the influence of different designs on the deformation of hydrogel sheets. fig. S6: the influence of pre-cross-linking on the cross-linking density gradient of hydrogels. fig. S7: the shape transformations of a helical hydrogel sheet. fig. S8: the 3D shape alteration of helical hydrogel sheets in mixed solutions. fig. S9: cooperative deformations. movie S1: the 3D deformation of a helical structure to show the stable structure in water after immersing in NaCl for 24 h and thorough washing with water. movie S2: the 3D deformation of a helical structure in the 0.1 M CaCl_2_ solution to demonstrate the reversible actuation after immersing in NaCl for 24 h and thorough washing with water.
