# Supplementary material for: Inside-Out 3D Reversible Ion-Triggered Shape-Morphing Hydrogels
Source: Research (Wash D C). 2019 Jan 14;2019:6398296. doi: 10.34133/2019/6398296 (PMC6750057; doi:10.34133/2019/6398296)
Supplement: Supplementary Materials — fig. S1: images of patterned silica wafer and sodium alginate hydrogel. fig. S2: the influence of Ca2+ ions concentration on the gelation process. fig. S3: investigation of the parameters involved in programmed deformations. fig. S4: mechanical properties of hydrogels. fig. S5: the influence of different designs on the deformation of hydrogel sheets. fig. S6: the influence of pre-cross-linking on the cross-linking density gradient of hydrogels. fig. S7: the shape transformations of a helical hydrogel sheet. fig. S8: the 3D shape alteration of helical hydrogel sheets in mixed solutions. fig. S9: cooperative deformations. movie S1: the 3D deformation of a helical structure to show the stable structure in water after immersing in NaCl for 24 h and thorough washing with water. movie S2: the 3D deformation of a helical structure in the 0.1 M CaCl2 solution to demonstrate the reversible actuation after immersing in NaCl for 24 h and thorough washing with water. [file 6398296.f1.zip › 6398296.f1/Figures.pptx]

## Slide 1
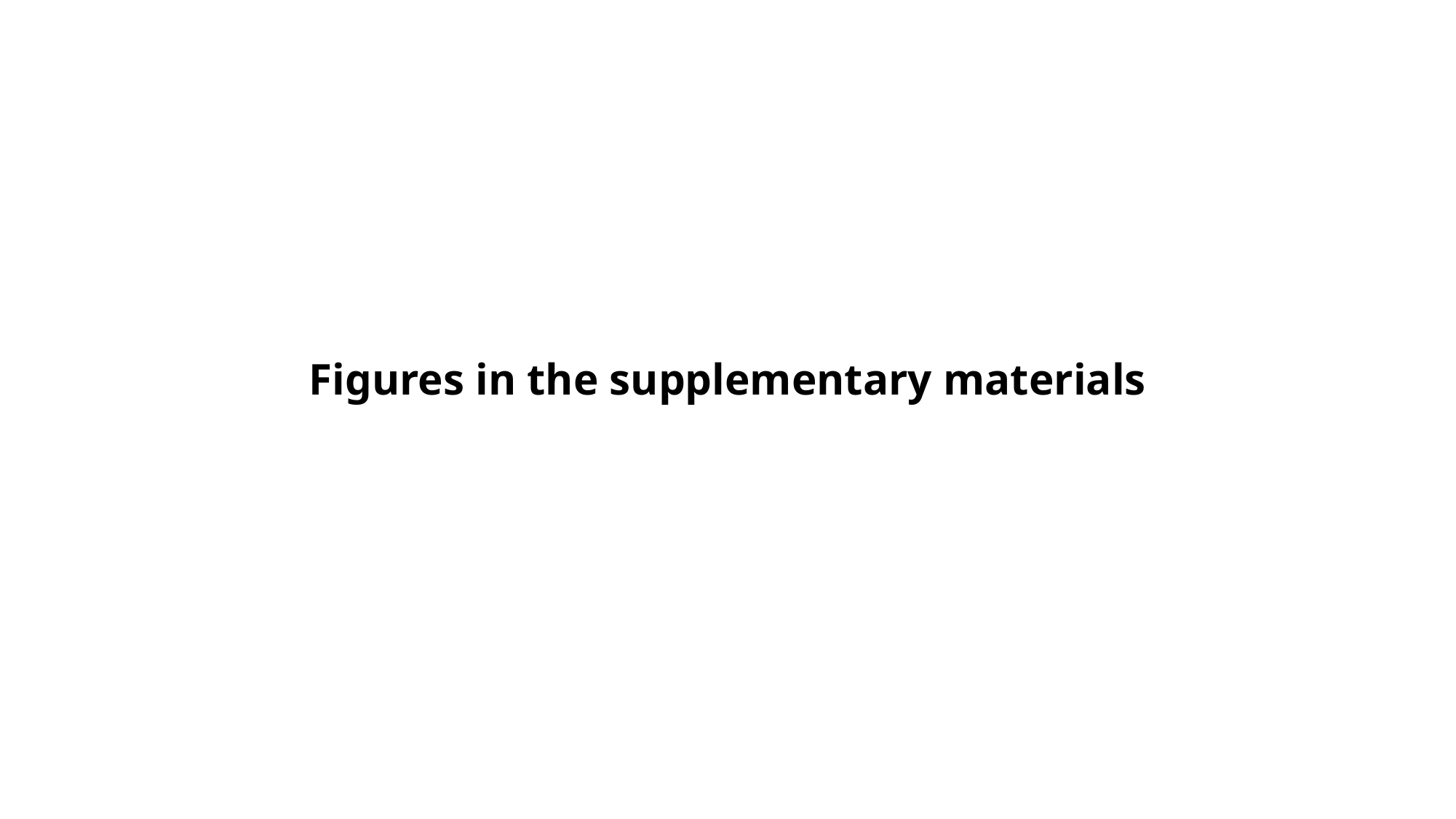

Figures in the supplementary materials

## Slide 2
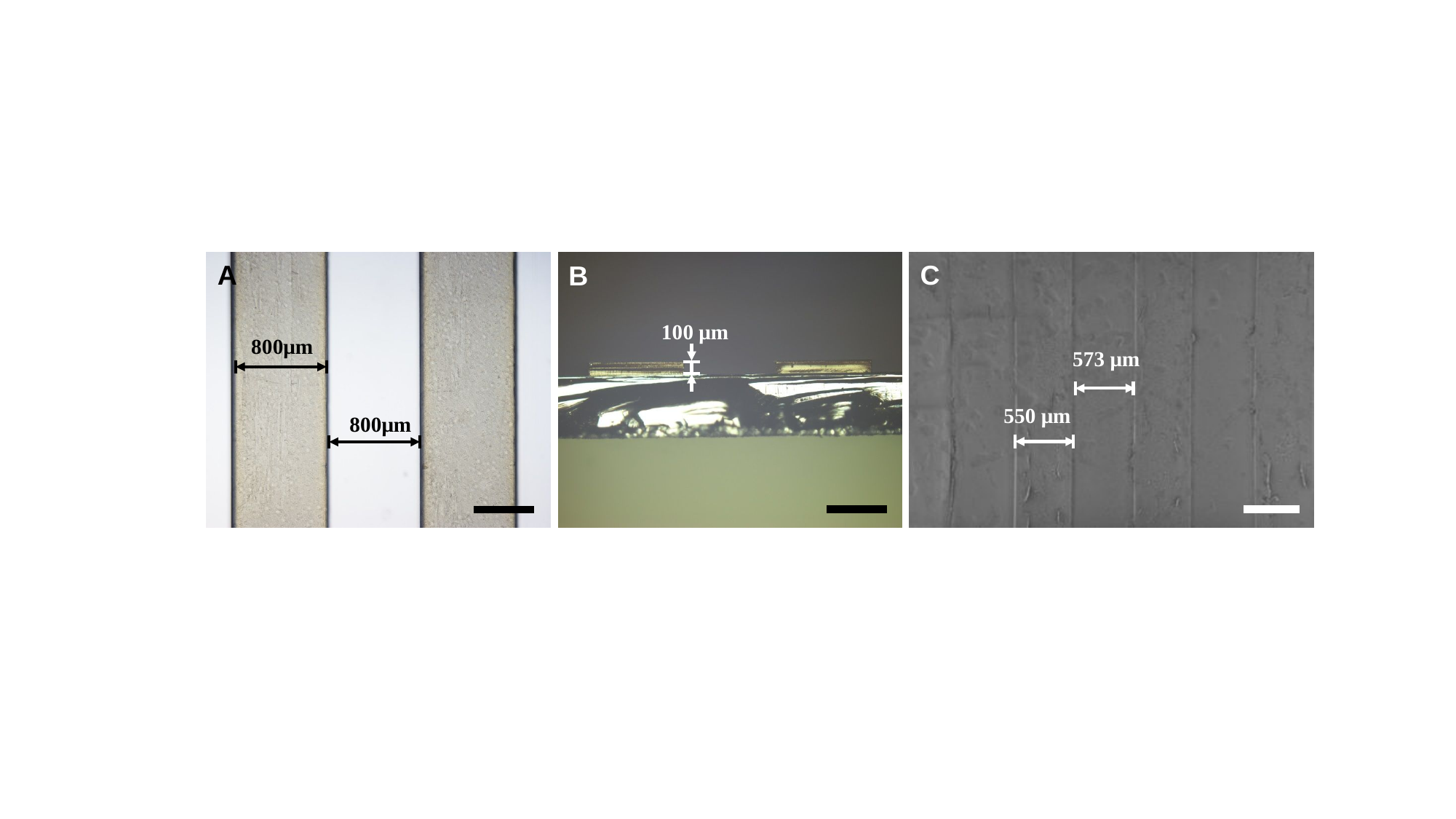

C
A
B
100 μm
800μm
573 μm
550 μm
800μm

## Slide 3
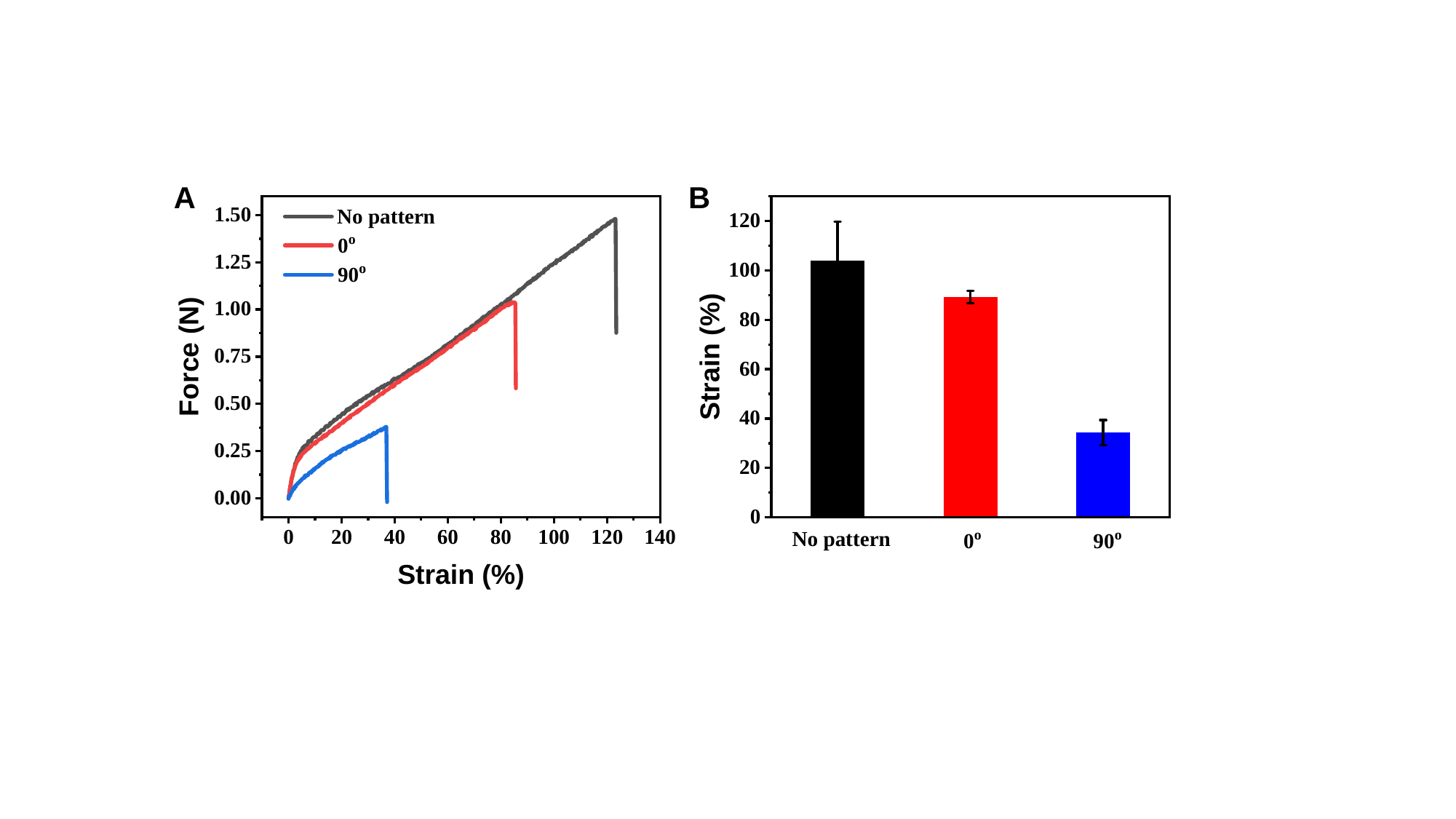

A
B

## Slide 4
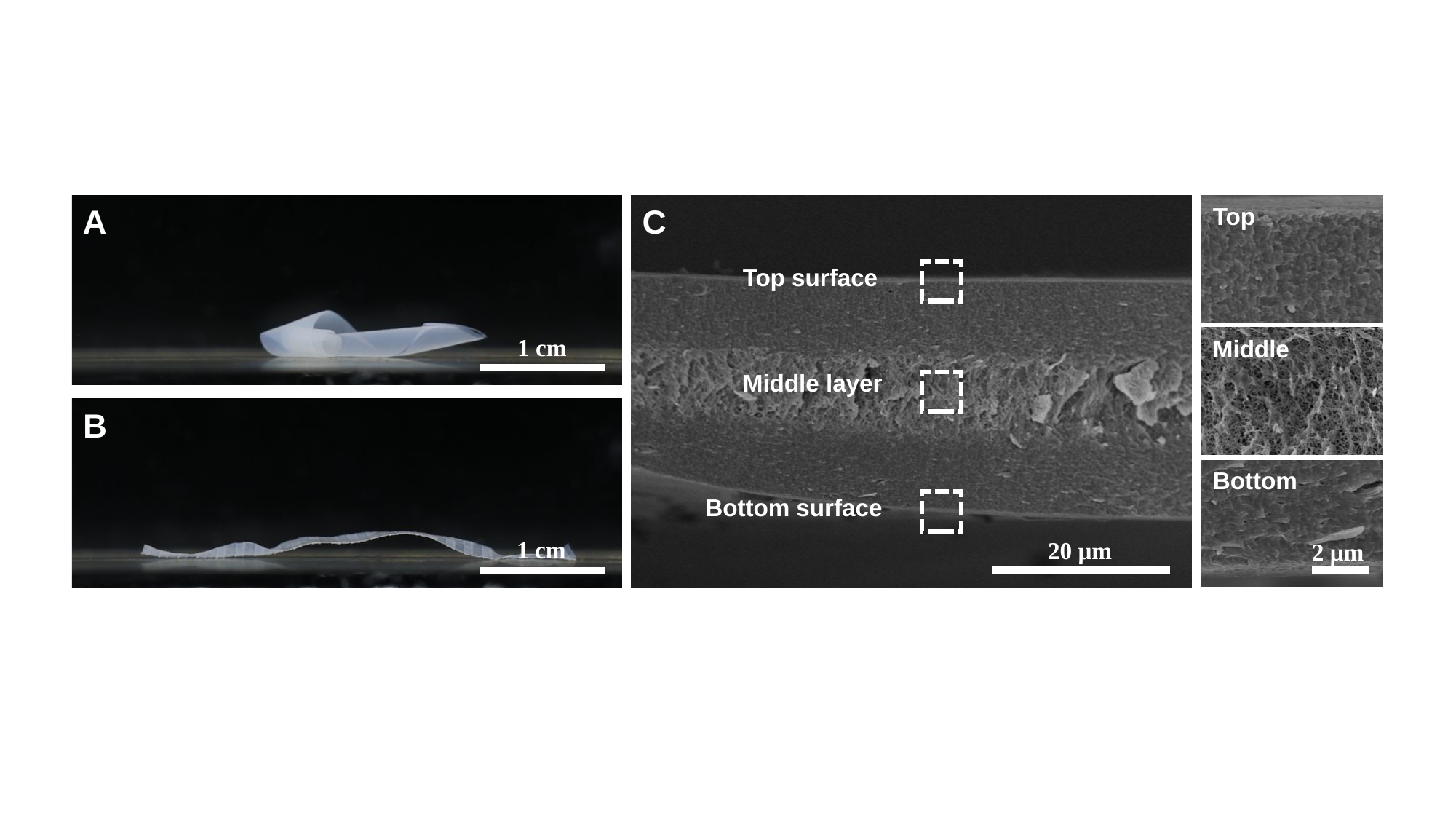

C
Top
A
Top surface
1 cm
Middle
Middle layer
B
Bottom
Bottom surface
1 cm
20 μm
2 μm

## Slide 5
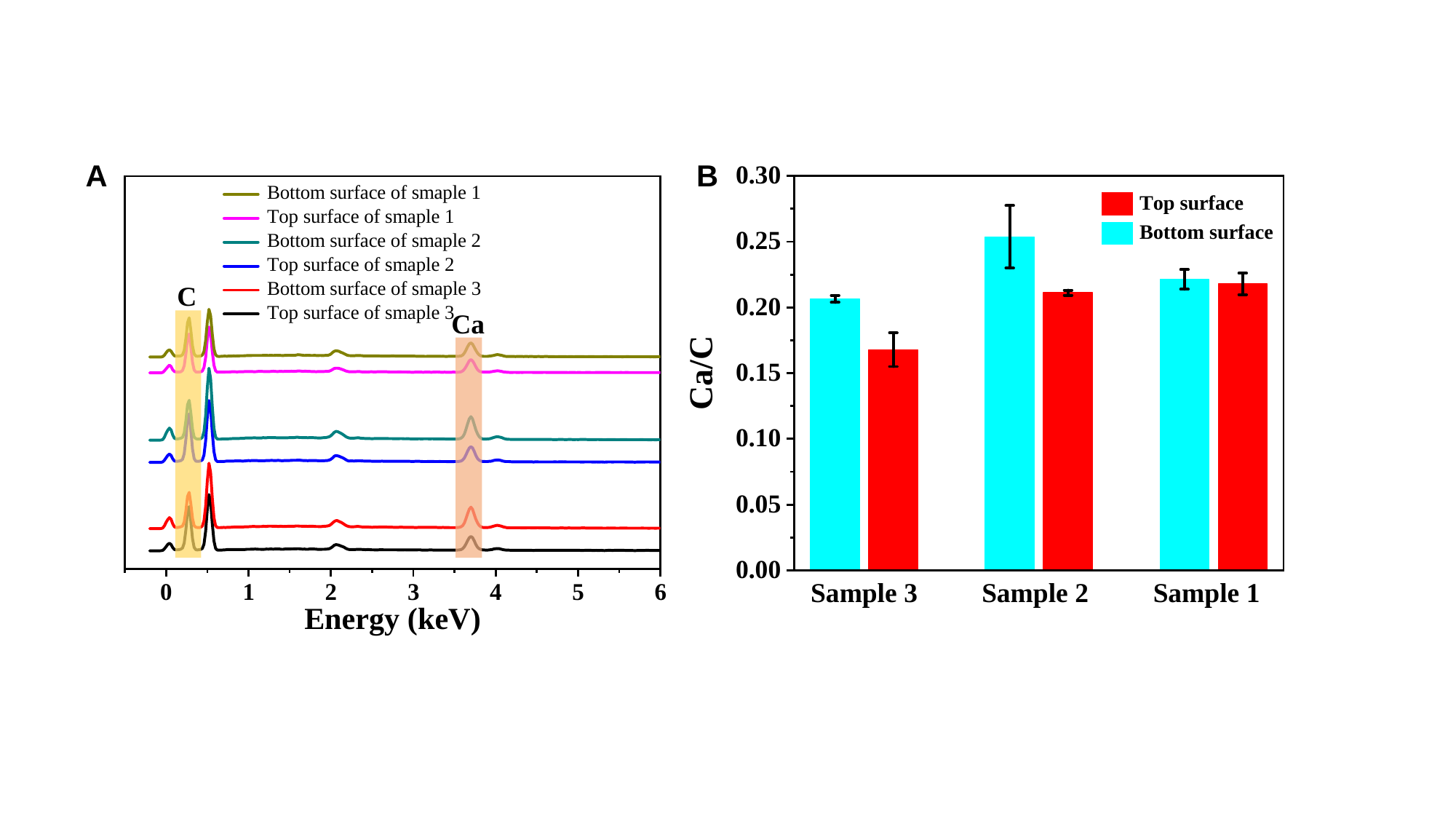

Sample 1
Sample 2
Sample 3
C
Ca
A
B

## Slide 6
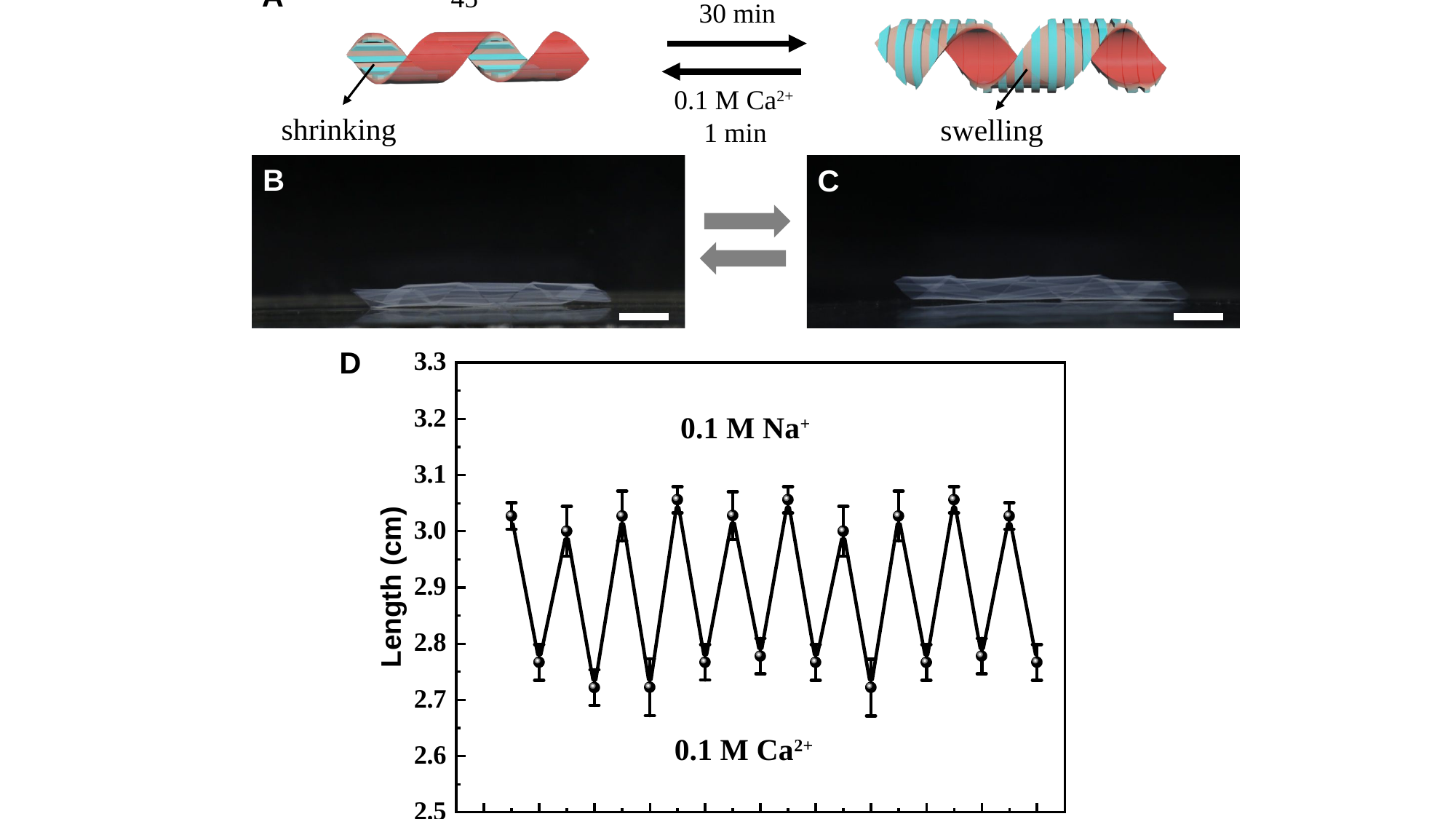

0.1 M Na+
30 min
A
45o
0.1 M Ca2+
1 min
shrinking
swelling
B
C
D
0.1 M Na+
0.1 M Ca2+

## Slide 7
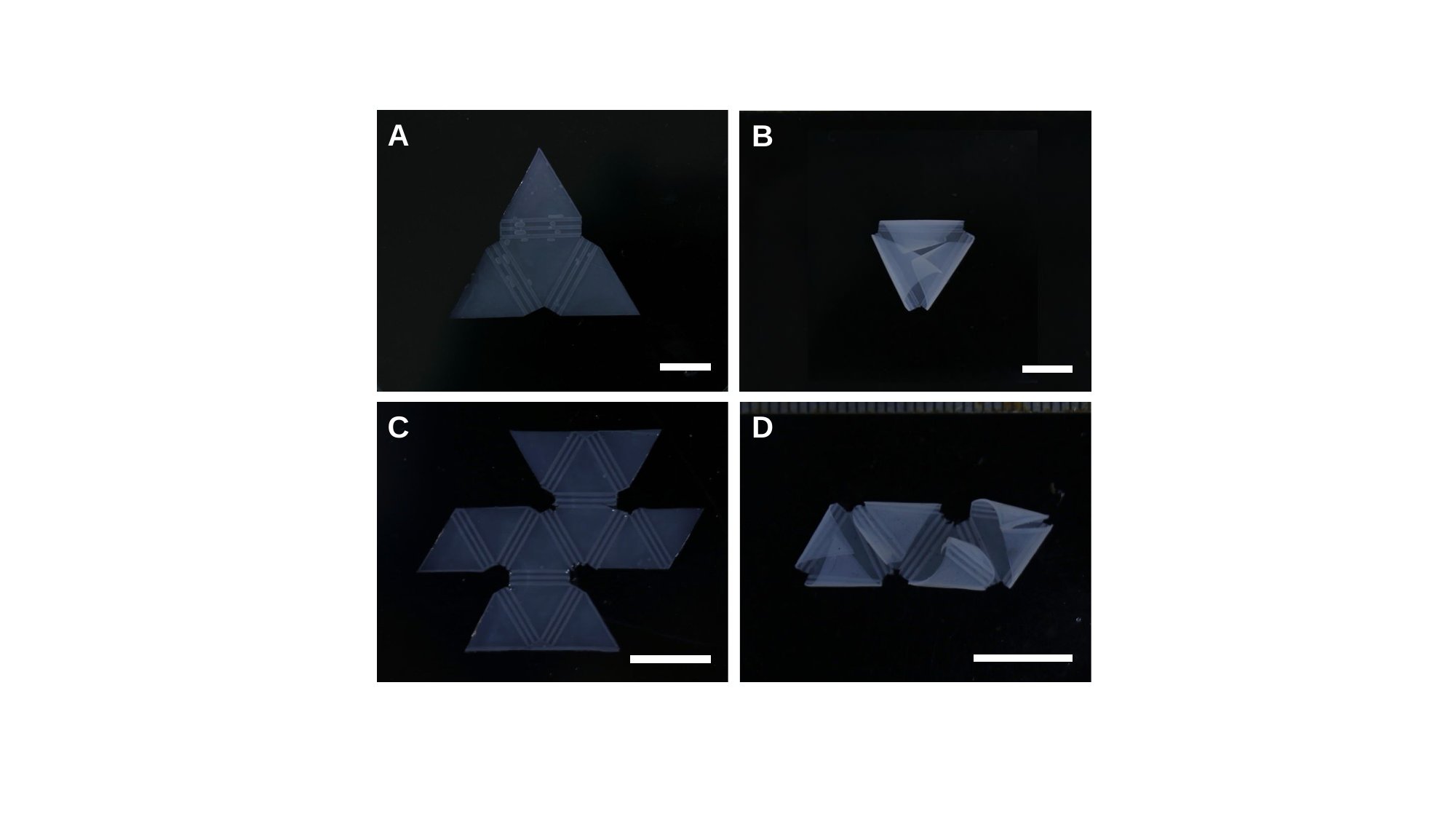

A
B
C
D
