# Supplementary figures and images for: Inside-Out 3D Reversible Ion-Triggered Shape-Morphing Hydrogels
Source: Research (Wash D C). 2019 Jan 14;2019:6398296. doi: 10.34133/2019/6398296 (PMC6750057; doi:10.34133/2019/6398296)

## Slide 1
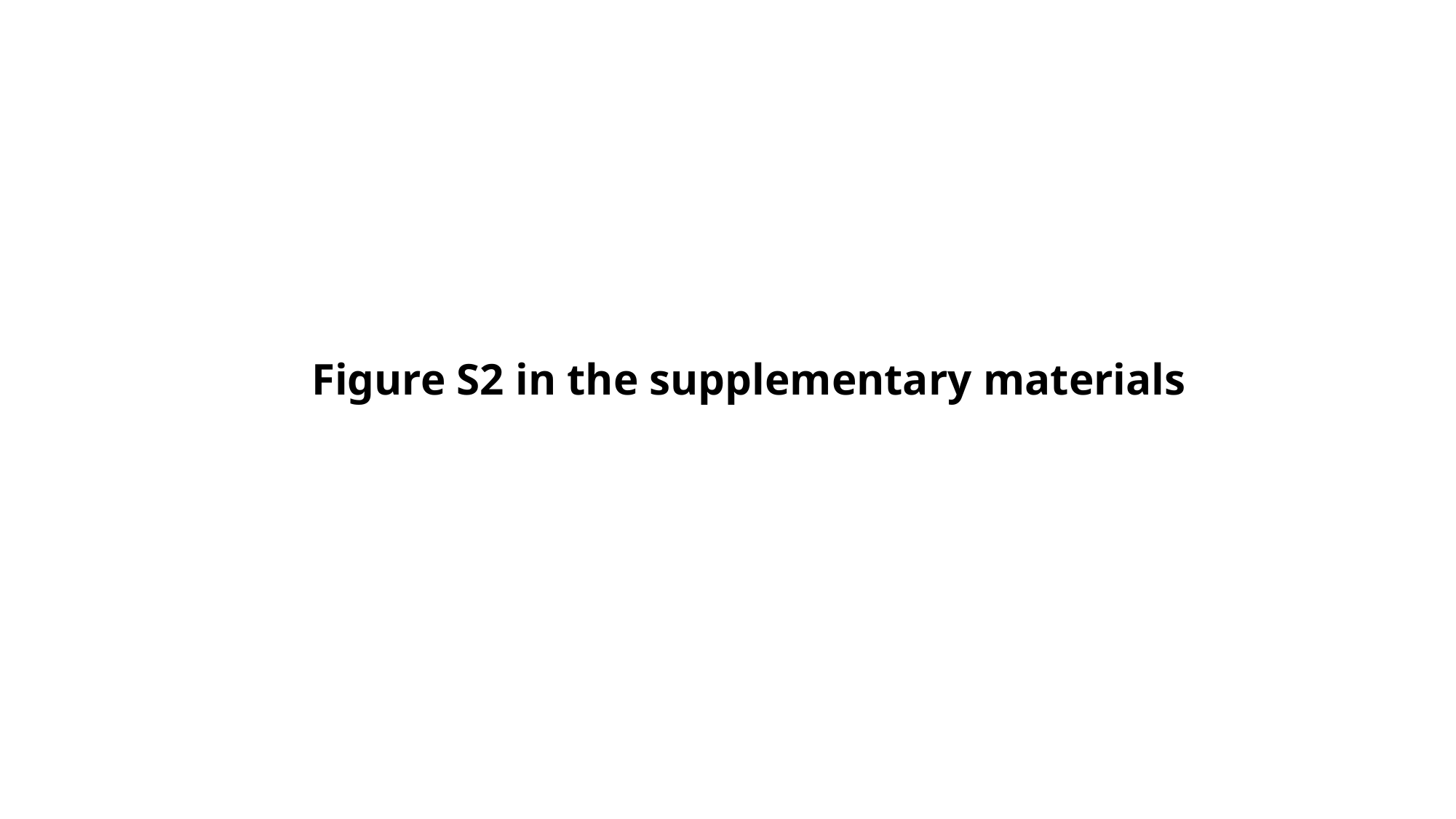

Figure S2 in the supplementary materials

## Slide 2
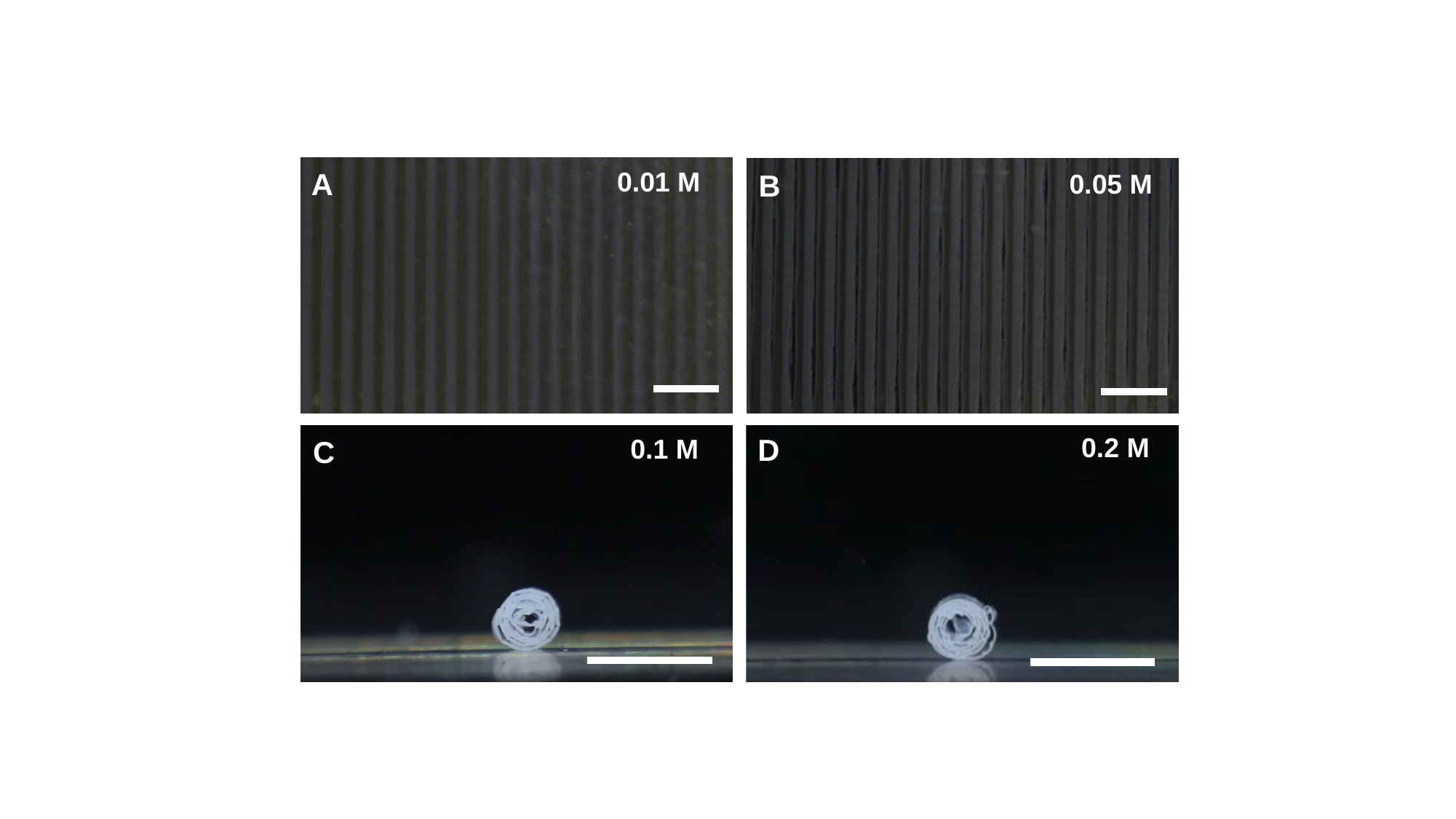

0.01 M
A
B
0.05 M
D
0.2 M
0.1 M
C

Supplement: Supplementary Materials — fig. S1: images of patterned silica wafer and sodium alginate hydrogel. fig. S2: the influence of Ca2+ ions concentration on the gelation process. fig. S3: investigation of the parameters involved in programmed deformations. fig. S4: mechanical properties of hydrogels. fig. S5: the influence of different designs on the deformation of hydrogel sheets. fig. S6: the influence of pre-cross-linking on the cross-linking density gradient of hydrogels. fig. S7: the shape transformations of a helical hydrogel sheet. fig. S8: the 3D shape alteration of helical hydrogel sheets in mixed solutions. fig. S9: cooperative deformations. movie S1: the 3D deformation of a helical structure to show the stable structure in water after immersing in NaCl for 24 h and thorough washing with water. movie S2: the 3D deformation of a helical structure in the 0.1 M CaCl2 solution to demonstrate the reversible actuation after immersing in NaCl for 24 h and thorough washing with water. [file 6398296.f1.zip › 6398296.f1/Figure S2.pptx]

## Slide 1
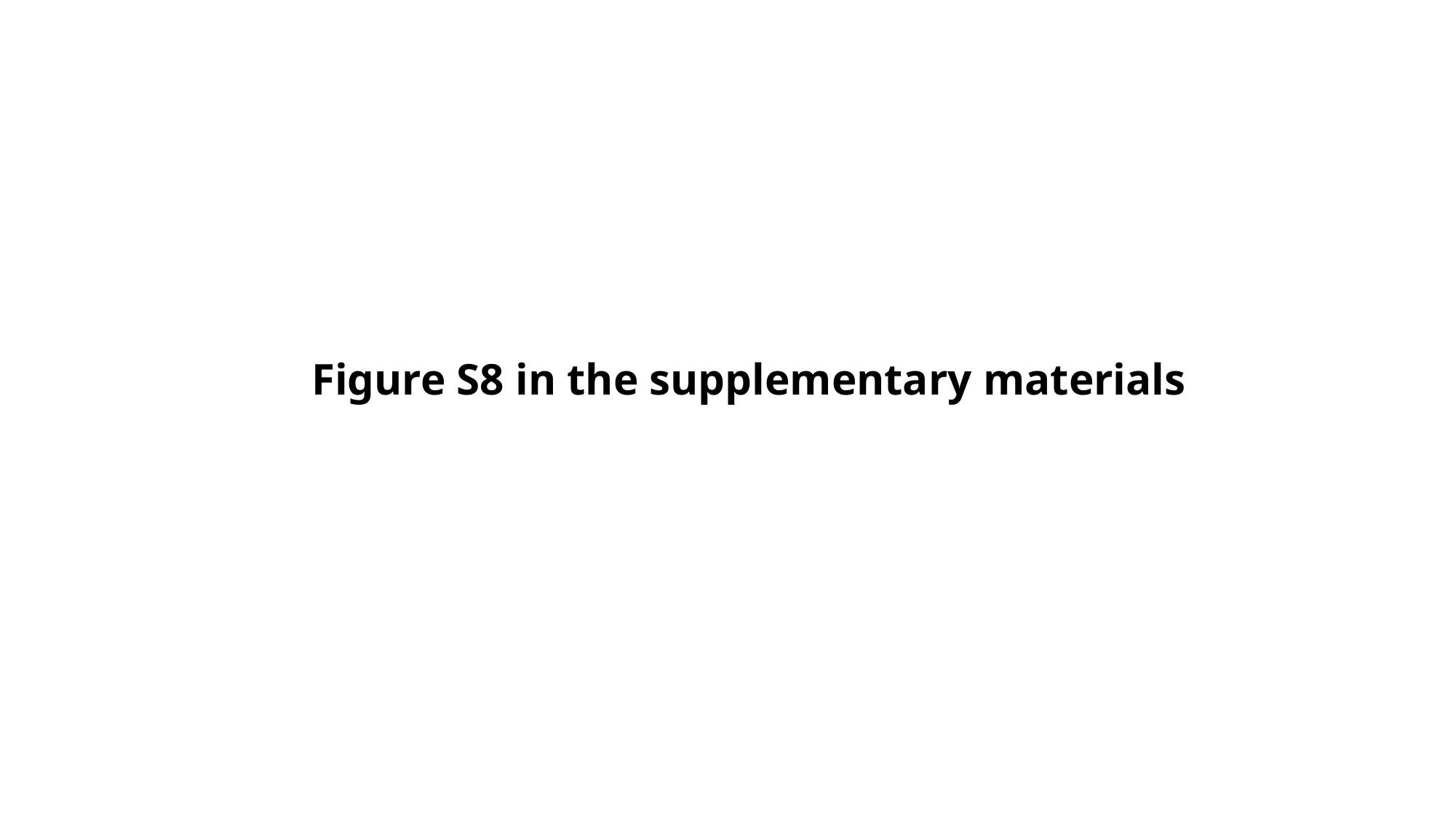

Figure S8 in the supplementary materials

## Slide 2
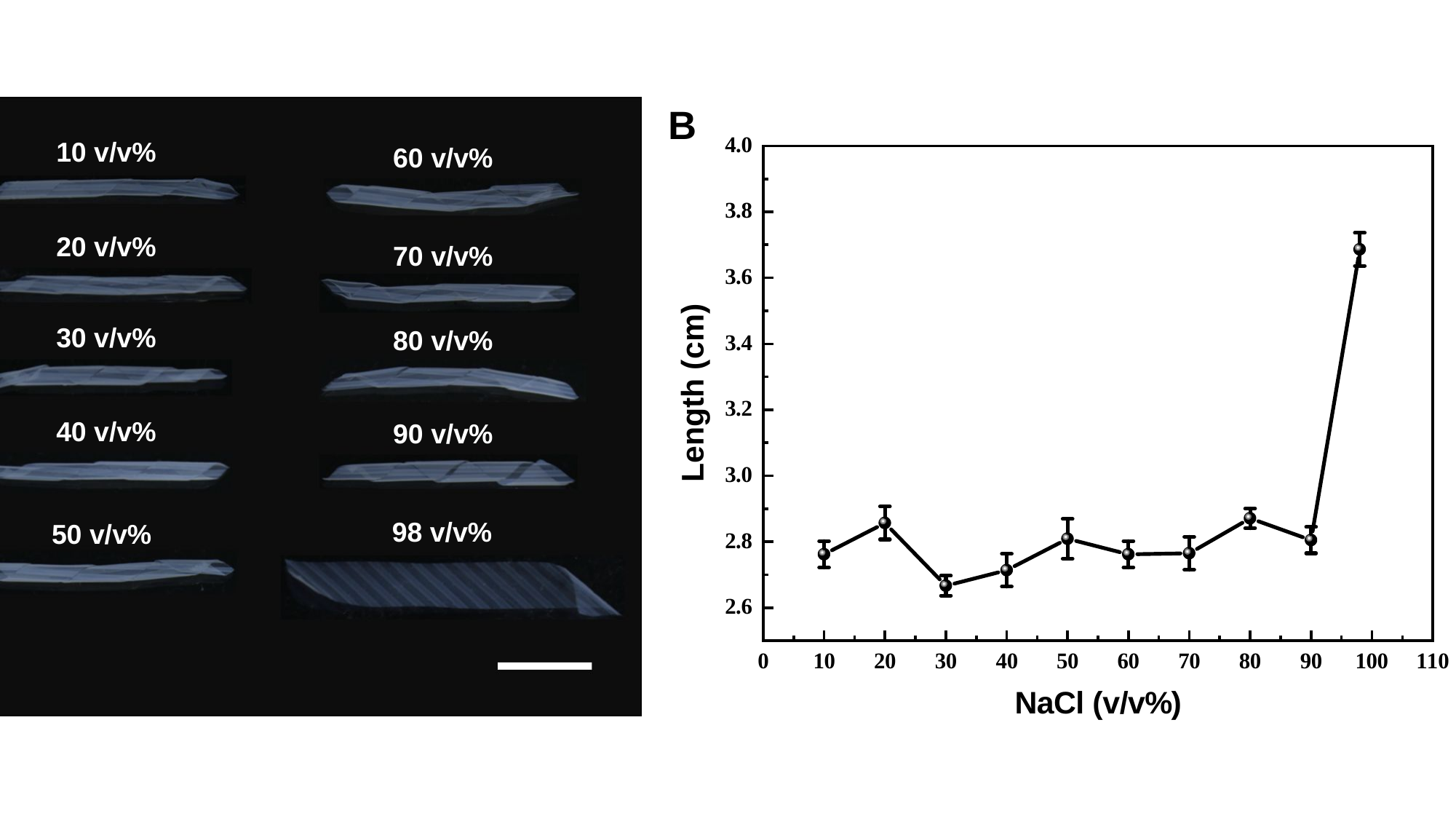

B
A
10 v/v%
60 v/v%
20 v/v%
70 v/v%
30 v/v%
80 v/v%
40 v/v%
90 v/v%
98 v/v%
50 v/v%

Supplement: Supplementary Materials — fig. S1: images of patterned silica wafer and sodium alginate hydrogel. fig. S2: the influence of Ca2+ ions concentration on the gelation process. fig. S3: investigation of the parameters involved in programmed deformations. fig. S4: mechanical properties of hydrogels. fig. S5: the influence of different designs on the deformation of hydrogel sheets. fig. S6: the influence of pre-cross-linking on the cross-linking density gradient of hydrogels. fig. S7: the shape transformations of a helical hydrogel sheet. fig. S8: the 3D shape alteration of helical hydrogel sheets in mixed solutions. fig. S9: cooperative deformations. movie S1: the 3D deformation of a helical structure to show the stable structure in water after immersing in NaCl for 24 h and thorough washing with water. movie S2: the 3D deformation of a helical structure in the 0.1 M CaCl2 solution to demonstrate the reversible actuation after immersing in NaCl for 24 h and thorough washing with water. [file 6398296.f1.zip › 6398296.f1/Figure S8.pptx]
